# The Hunt for Degrons of the 26S Proteasome

**DOI:** 10.3390/biom9060230

**Published:** 2019-06-13

**Authors:** Hadar Ella, Yuval Reiss, Tommer Ravid

**Affiliations:** Department of Biological Chemistry, Institute of Life Sciences, the Hebrew University of Jerusalem, Jerusalem 91904, Israel; ella@mail.huji.ac.il (H.E.); yuvalr@mail.huji.ac.il (Y.R.)

**Keywords:** ubiquitin-proteasome system, degrons, E3-ubiquitin ligases, protein quality control, high throughput screens, next generation sequencing

## Abstract

Since the discovery of ubiquitin conjugation as a cellular mechanism that triggers proteasomal degradation, the mode of substrate recognition by the ubiquitin-ligation system has been the holy grail of research in the field. This entails the discovery of recognition determinants within protein substrates, which are part of a degron, and explicit E3 ubiquitin (Ub)-protein ligases that trigger their degradation. Indeed, many protein substrates and their cognate E3′s have been discovered in the past 40 years. In the course of these studies, various degrons have been randomly identified, most of which are acquired through post-translational modification, typically, but not exclusively, protein phosphorylation. Nevertheless, acquired degrons cannot account for the vast diversity in cellular protein half-life times. Obviously, regulation of the proteome is largely determined by inherent degrons, that is, determinants integral to the protein structure. Inherent degrons are difficult to predict since they consist of diverse sequence and secondary structure features. Therefore, unbiased methods have been employed for their discovery. This review describes the history of degron discovery methods, including the development of high throughput screening methods, state of the art data acquisition and data analysis. Additionally, it summarizes major discoveries that led to the identification of cognate E3 ligases and hitherto unrecognized complexities of degron function. Finally, we discuss future perspectives and what still needs to be accomplished towards achieving the goal of understanding how the eukaryotic proteome is regulated via coordinated action of components of the ubiquitin-proteasome system.

## 1. Introduction

The maintenance of cellular protein homeostasis (proteostasis) is a vital mechanism in all organisms, which is largely governed by the adjustment of intracellular protein levels to the cell needs. This is achieved by fluctuating protein turnover rates, determined by the ratio between the rates of protein synthesis and degradation. Proteolysis, a major mechanism of the intracellular proteostasis guardian network, is primarily executed by the ubiquitin (Ub)-proteasome system (UPS) [1]. Within this system, the half-life time of proteins is determined by selective interaction of proteins with specific E3 Ub-protein ligases, the substrate recognition modules of the UPS [2]. Understanding the mode of substrate recognition by E3 ligases has been of high interest for UPS researchers since its discovery, more than four decades ago [3]. This continuous effort led to the identification and the characterization of multiple degradation signals, coined degrons [4].

Degrons can be generally divided into two major groups, based on the characteristics of the signal that triggers degradation. Inherent degrons, permanently present in proteins and acquired degrons that are induced by post translational modifications (PTMs). Inherent degrons can be specific amino acid sequences, such as the destruction box of cyclins [5], or N- and C-terminal amino acids corresponding to the N-degron and the C-degron pathways [6]. In addition, a large portion of inherent degrons consists of hydrophobic sequences, normally buried in the protein core, or within interaction surfaces between subunits of protein complexes. These degrons are frequently exposed when proteins fail to fold properly, upon protein misfolding or when protein complexes fail to assemble [7,8,9,10]. The exposure of hindered degrons can be induced by multiple mechanisms, including changes in the environmental or intracellular conditions as well as by genomic mutations. These perturbations initiate a protein quality control (PQC) response that either assists misfolded protein re-folding or triggers their elimination by the UPS [11].

Unlike inherent degrons, acquired degrons are transient elements obtained via PTMs, such as phosphorylation, ligation of Small Ubiquitin-like Modifier (SUMOylation) and hydroxylation [12,13,14]. Protein phosphorylation is by far the most common PTM that triggers ubiquitylation via E3 Ub ligases of the Skp, cullin, F-box (SCF) containing complex, also termed cullin-RING ligases (CRLs) [15]. Recognition of transient protein phosphorylation allows rapid degradation and is hence prevalent in pathways that require abrupt fluctuation in protein levels such as cell-cycle regulation. For example, Sic1, an inhibitor of G1/S transition, is recognized by the Ub ligase SCF^Cdc4^ only when phosphorylated, while the phosphorylation of the G1 cyclin, Cln2, determines recognition by the Ub ligase SCF^Grr1^ [16,17]. Occasionally, recognition of degradation substrates requires initial activation of their cognate E3 ligase, such as in the case of the neddylation of cullins, the scaffold components of the SCF family members [18] or the activation of APC/C and parkin RING E3 ligases via phosphorylation [19,20].

Substrate recognition by E3 ligases is considered as the rate-limiting step of the UPS degradation machinery. This key step was initially detected by A. Hershko and co-workers, following the discovery of the Ub system, using Ub affinity chromatography approach for isolating prototype enzymes of the Ub conjugation system [3,21]. In addition to fractions required for the formation of high energy Ub-enzyme thiol bond, for which E1 and E2 were required, transfer of Ub to proteins required a third enzyme, consequently termed E3, that consists the substrate binding site of the Ub ligation system. This finding was based on strict linkage between the ability of a substrate to bind to an E3 and the rate of Ub conjugation and degradation [22]. Furthermore, a correlation was established between the properties of the amino terminus of a substrate and its ability to bind to the E3 [22]. Consequently, the identification of N-end degrons enabled the isolation by affinity chromatography of the first E3, termed E3α, the enzyme that recognizes proteins with basic or bulky hydrophobic α-amino group at their amino terminal residue [23]. This mode of substrate recognition, later dubbed as the N-end rule, was thoroughly investigated and extended in yeast by Varshavsky and colleagues, [24,25], and was recently re-named the N-degron pathway [6].

In subsequent years, many degron features, mostly of the acquired type, have been identified [12]. These degrons are frequently randomly discovered when regulatory mechanisms of cellular pathways are under study. However, the repertoire of known degrons is still very limited and their presence obviously cannot explain the vast variability in the half-life times of the bulk of cellular proteins. The major obstacle in identifying novel degrons is that they cannot be deduced from the features of already known degrons and consequently, there are no guidelines for a methodical degron search.

The emergence of high throughput genomic methods has provided a powerful unbiased means to identify the entire landscape of cellular degrons (degronome) and thus, to decipher rules that govern the instability of the proteome. This review describes key degron identification endeavors, undertaken since the discovery of the UPS, aimed at identifying inherent degrons, and the contribution of these efforts to the understanding of how cellular proteostasis is regulated.

## 2. Principles and Progression of Degron Discovery

Inherent degrons are an integral feature of proteins that can confer instability when appended to otherwise unrelated stable proteins. The autonomous function of degrons, has made the use of stable reporter-degron fusion proteins the mainstay of degron discovery systems, which is the focal point of this review. All unbiased degron search methods are based on the ability of a degron to destabilize an otherwise stable reporter. Stability assays vary, depending on the type of the reporter—growth assays are applied for reporters that are metabolic markers while colorimetric and fluorescent measurements are employed for enzyme and fluorescent reporter proteins (respectively).

### 2.1. Colorimetric Selection

**Figure 1:**
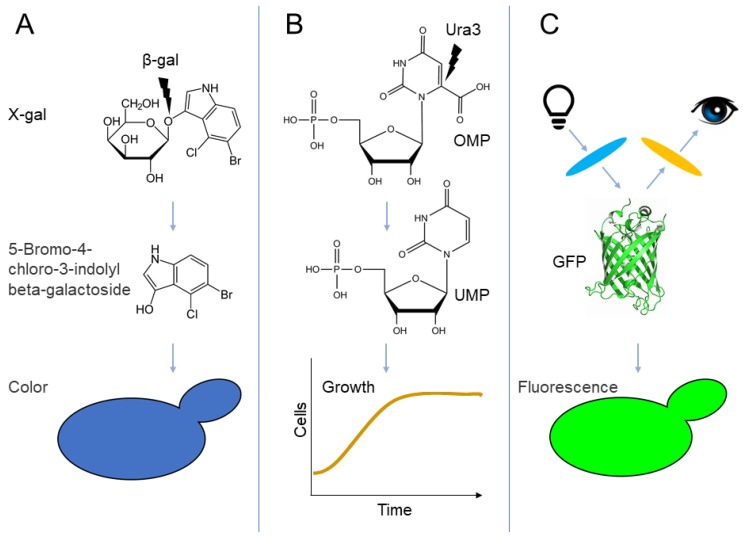
Reporter systems for degron discovery. (**A**) β-gal hydrolyzes X-gal to produce 5-bromo-4-chloro-3-indolyl β-galactoside is detected by formation of dark blue-colored yeast colonies. (**B**) Orotidine-5′-phosphate decarboxylase (Ura3) catalyzes the decarboxylation of OMP, converting it into UMP, an intermediate of pyrimidine biosynthesis. Ura3 activity is essential for growth of uracil auxotroph yeast. (**C**) Excitation of green fluorescent protein (GFP) causes the emission of fluorescence signal, visualized by fluorescence microscopy. OMP: orotidine 5′-monophosphate; UMP: uridine 5′-monophosphate.

**Figure 2:**
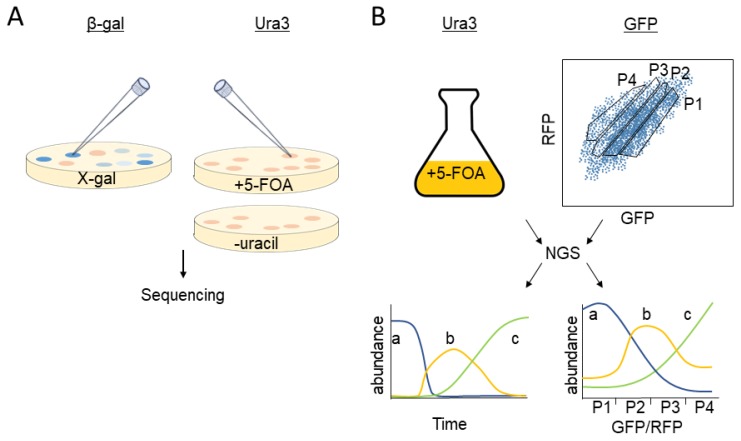
Methods for degron discovery. (**A**) Colony isolation on solid media. Left, yeast mutants, defective in β-gal-degron degradation are detected by dark blue appearance of yeast colonies. Right, degrons in Ura3-expressing cells are detected by the ability of yeast cells to grow on plates containing the orotic acid derivative 5-FOA or the inability to grow in the absence of uracil. (**B**) Isolation of degron from cells grown in liquid media. Left, degrons in Ura3-expressing yeast cells are isolated from cells after prolonged growth period in the presence of 5-FOA. The degron potency correlates with its abundance in the yeast population so that the frequency of stronger degron increases with time. Right, cells expressing fluorescence markers are divided by fluorescence-activated cell sorting (FACS) into separated bins, based on their cellular GFP/RFP ratio. Strong degrons are present in bins with low GFP/RFP ratio (P4). a—no degron; b—intermediate degron; c—strong degron. 5-FOA: 5-fluoroorotic acid; NGS: next generation sequencing; RFP: red fluorescent protein.

Colorimetric selection through use of β-Galactosidase (β-Gal), the product of the *Escherichia Coli LacZ* gene, is a well-established method to study biological processes [26]. Initially, the enzyme was used for UPS research as a reporter, to determine the influence of amino-terminal residues on protein stability [27] and to identify destabilizing regions within the yeast transcription factor Matα2 and isolate degradation-defective mutants [28]. The enzyme β-Gal hydrolyses the lactose analog X-gal, producing a blue color and thus, yeast expressing the enzyme form blue colonies, when grown in the presence of the sugar [29] (Figure 1A). Accordingly, when β-Gal is fused to a degron, enzyme levels diminish, resulting in reduced X-gal hydrolysis, indicated by a pale blue to white appearance [28]. Involvement of Ub-mediated degradation can be confirmed upon reversal of β-Gal instability (or any reporter for that matter) in yeast cells where the cognate UPS pathway is defective [28] (Figure 2A). The simplicity of the colorimetric assay provided the incentive for several research attempts to utilize the β-Gal enzyme as a reporter in screens aimed at identifying novel degrons [30,31].

### 2.2. Growth Selection

A significant improvement in degron research was achieved when growth selection markers were introduced as reporters and substituted the colorimetric assays. The coupling of cell growth to degron activity is more sensitive, has a wider dynamic range, present a clear starting reference and above all, can be accurately quantified. A common yeast selection marker is the product of the *URA3* gene, the enzyme Orotidine-5′-phosphate decarboxylase (Ura3). The enzyme is essential for pyrimidine ribonucleotide biosynthesis [32] (Figure 1B) and therefore, low steady-state levels of Ura3 reduces growth of auxotrophic yeast cells on minimal medium lacking uracil [33] (Figure 2A). Accordingly, when Ura3 is attached to a degron, cell growth in the absence of uracil is inhibited. To adapt Ura3-based selection to degron screen, a positive selection method had been devised, where Ura3 degradation confers growth advantage. This was achieved by addition 5-fluoroorotic acid (5-FOA) to the culture medium (Figure 2A). Since 5-FOA is converted by Ura3 to the toxic compound 5-fluorouracil, enhanced Ura3 degradation confers growth advantage [34]. Thus, in order to identify degrons in yeast, Kulka and co-workers appended a truncated genomic library downstream to the *URA3* gene and subsequently compared between growth of yeast cells on 5-FOA-containing or uracil-deficient agar plates [31]. Subsequently, degron recognition was assigned to a specific E3 ligase through a secondary screen where potential degrons were expressed in cells lacking the Doa10 cognate E2 enzymes, Ubc6 and Ubc7. Employing this screening method, the Kulka Lab identified nine degrons, compared to only one discovered by the β-Gal assay [31]. One of these degrons, termed *CL1*, has since been used as a model degron for proteasomal degradation in both yeast and mammals [35,36].

Almost two decades later, improved screening and sequencing techniques enabled Michaelis and co-workers, to identify ten-fold more degrons, using the Ura3-based growth selection as readout [37]. These degrons were classified based on a correlation between growth kinetics and distinct sequence and hydrophobicity criteria and were assigned to distinct Ub-conjugation pathways. Another key finding of this study was the absolute requirement for the heat shock protein 40 (Hsp40) chaperone Ydj1 and either of the Hsp70s Ssa1 or Ssa2 for the degradation of all tested degrons, suggesting that these heat shock family members are invariable components of PQC-associated degradation in yeast.

Despite several improvements, the Michaelis degron search was still limited to single colony selection and hence, the number of degrons that it produced was too small to draw comprehensive structure-function correlations or to gain other conclusive biological insights. Obviously, hunting for something unpredictable and potentially highly diverse requires high throughput (HTP) research systems. These requirements were addressed in our lab through the development of a method dubbed GiLS (Growth kinetics in Liquid culture under Selective conditions) [38] and applying it to measure Ura3-dependent growth in the presence of 5-FOA [39]. In brief, a complementary DNA (cDNA) library was appended to the 3′ end of Ura3 in all three possible open reading frames, in order to maximize the probability of identifying authentic degron sequences. Consequently, growth rate in the presence of 5-FOA revealed Ura3-degrons and their potency—the faster the replication the stronger the degron. Practically, the frequency of Ura3-degron clones increased until they took over the entire yeast population (Figure 2B, left panel). Indeed, thousands of DNA sequences, isolated from cultures after a 48-h growth period, were confirmed as degrons [39].

### 2.3. Direct Determination of Fluorescent Reporter Levels

Selection in liquid media is an efficient method to detect degrons. Yet, it entails a cumbersome routine that requires constant culture dilution and multiple samplings. The introduction of fluorescence-activated cell sorting (FACS) by flow cytometry has simplified global degron searches, since it can measure a protein steady-state level without selection by single read of a fluorescent-reporter (Figure 2B, right panel). Moreover, FACS-based degron screens are advantageous over degron selection in liquid media not only because the data acquisition is simpler but also because cell populations can be subjected to multiple analyses, simultaneously or in tandem, thus allowing acquisition of comprehensive data without the requirement for complementary screens.

**Figure 3:**
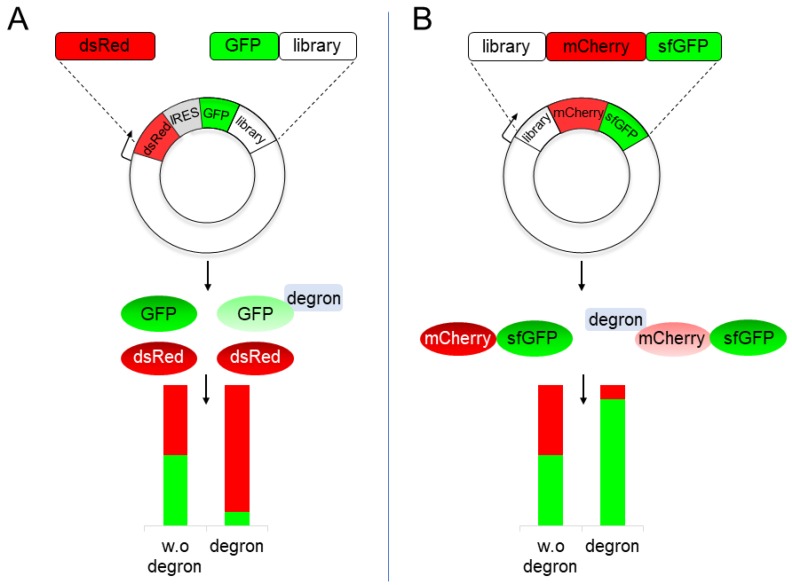
High throughput (HTP) degron screens using a GFP reporter. (**A**) The global protein stability (GPS) cassette consists of an internal ribosome entry site (IRES), flanked by Dicscosoma red fluorescent protein (dsRed) and a GFP-library coding sequences, all of which are transcribed from a single promoter albeit translated separately. Reduced GFP/RFP indicates destabilization of the GFP, due to the presence of a degron. (**B**) Tandem fluorescent timers (tFT) cassette consists of library-mCherry-sfGFP fusion. Reduced mCherry/sfGFP ratio is attributed to the presence of a degron that shorten the half-life of the chimera protein. mCherry: monomeric Cherry; sfGFP: super folder GFP; w.o: without.

All non-selective, fluorescence-based, methods employ jellyfish *Aequorea victoria* green-fluorescent protein (GFP) as a degron reporter. Initially, Hampton and co-workers employed a GFP reporter to explore changes in the steady-state levels of yeast hydroxymethylglutaryl CoA reductase 2 (HMG2), the rate-limiting enzyme of sterol biosynthesis [40]. However, metabolic regulation may affect both the rates of synthesis and degradation of HMG2, a distinction that a simple flow cytometry-based quantification cannot discern. To address this shortcoming, the Elledge Lab devised a bimodal fluorescent expression cassette termed Global Protein Stability (GPS) [41]. The GPS cassette consists of an internal ribosome entry site (IRES) flanked by Dicscosoma red fluorescent protein (dsRed) and GFP coding sequences, all of which are transcribed from a single promoter. As a result, both fluorescent proteins are produced in equal stoichiometry [42]. Hence, when a degron is fused to GFP, the effect of protein degradation can be accurately determined by the steady-state ratio of GFP/dsRed—the lower the ratio, the less stable GFP is (Figure 3A). Through GPS, multiple substrates of RING Ub ligases were identified [43], including human carboxyl-terminus operating degrons [41].

Another FACS-based method for direct measurement of protein stability has been developed at the Knop lab. The reporter system, termed tandem fluorescent protein timers (tFT), employs a vector that expresses mCherry, fused at the C-terminus to a superfolder variant of GFP (sfGFP), resulting in the expression of a mCherry-sfGFP chimera protein (Figure 3B). Due to a much faster sfGFP folding, its GFP fluorescent emission precedes that of mCherry, allowing the measurement of mCherry-sfGFP stability through determination of the mCherry/sfGFP fluorescence ratio—the lower it is, the less stable the chimera is [44]. The tFT method was employed to identify substrates of the Asi E3 Ub-protein ligase of the inner nuclear envelop [45] as well as N-terminally operating degrons [46].

### 2.4. Yeast-2-Hybrid-Based Screening

Yeast-2 hybrid (Y2H) is a common high throughput method for studying protein-protein interactions [47]. Since E3 Ub ligases are the substrate binding module of the Ub ligation system [2], Y2H was employed in UPS research to discover E3-specific degrons. Y2H-screening relies on the mode of action of the transcription factor Gal4, that stimulates transcription by binding to an upstream activation sequence (UAS). DNA binding and transcription activation functions are assigned to independent binding (BD) and activation (AD) domains, that even when expressed in separate proteins can associate and drive transcription. Accordingly, in order to identify PQC substrates of the yeast nuclear E3 San1, a RING domain mutant was fused to Gal4BD and served as bait for a random library of ∼10^7^ 16-mer peptides, fused to Gal4AD [7]. The assay was designed so that gene transcription, indicative of peptide-E3 binding, resulted in growth on selective medium. Consequently, the screen detected ten peptides, all exhibiting hydrophobic sequences, likely to be part of a San1 recognition determinant. The Y2H method is most suitable for detecting E3-specific degrons when there is a direct interaction between the two. The drawback is that Y2H cannot be applied for global degron discovery where the cognate E3 ligase is unknown.

### 2.5. Design of DNA Libraries for Degron Discovery

A major challenge of degron discovery is to identify authentic degrons, that is, polypeptide sequences that are part of real intracellular proteins. With that goal in mind, the pioneering Geffen study employed a cDNA library joined to the Ura3 3′ end in three different reading frames [39]. Thus, not only were the sequences derived from open reading frames of expressed cellular proteins, but the design also ensured that at least one sequence was in an authentic protein reading frame. Indeed, the improved screen and library construction methods revealed thousands of putative degron sequences, hundreds of which were derived from authentic yeast proteins.

Yet, cDNA libraries are still not optimal for degron mining because they are mirrors of the transcriptional status at the time they are generated. Hence, they do not represent the entire genome, far from it, many sequences are either over or under-represented or absent altogether. This drawback has been solved by the emergence of state-of-the-art oligonucleotide synthesis that generates custom-made oligonucleotides, encoding accurate proteome sequences. The advantage of these libraries was demonstrated by recent degron discovery projects that employed synthetic DNA libraries covering the entire eukaryotic proteome, including full-length and tiled human peptidome libraries [41,48] and human C-terminal and yeast N-terminal libraries [41,46]. Interestingly, accidental errors in oligonucleotide synthesis produced sequences of aberrantly terminated proteins that exposed C-terminal residues that were otherwise cryptic. This erroneous byproduct led to the discovery a novel PQC mechanism at C-termini of abnormal proteins, regulated by CRL-type E3 Ub-protein ligases [48], demonstrating that not every sequencing error is actually bad for research.

### 2.6. Data Acquisition and Processing

Improved degron screens, employing either growth- or FACS-based methods, identify populations of degron-expressing cells rather than single colonies. Obviously, identifying degrons-encoding DNA sequences of a given cell population, requires a substantial DNA sequencing capacity, a feat facilitated through next generation (NGS) Sequencing [49]. Initially developed for the sequencing of the human genome [50,51], NGS determines the sequence of hundreds of millions of DNA fragments in a single run. NGS was used by Stable-seq, a method employed to screen a library of ~30,000 *Deg1* degron mutants, in order to identify critical amino acid sequence elements [52]. To this end, the mutant *Deg1* degron library had been fused the metabolic marker Leu2, after which cells were selected for growth in the absence of leucine. The screen thus isolated clones where Leu2 had been stabilized by an abrogated degron to various degrees that were subsequently quantified by NGS sequencing.

While NGS provides the relevant data, bioinformatic software makes sense out of it. It discerns enrichment or depletion of various sequences [53], common features within sequences [54], and predicts secondary structures [55]. Consequently, since Stable-Seq introduction, NGS sequencing followed by application of powerful bioinformatic algorithms for data analysis have become the mainstay of HTP degron discovery investigations [39,41,46,48]. A typical workflow of a high throughput degron discovery project is presented in Figure 2B.

## 3. Identification of Novel Degrons and their Cognate E3 Ligases

Degrons are *cis*-acting elements that are recognized by E3 Ub-protein ligases [1]. Therefore, both degron discovery and subsequent identification of cognate E3 ligases are complementing missions that must be accomplished in order to understand both temporal and spatial regulation of proteostasis. Indeed, combined HTP screens that identify both degrons and their cognate E3 ligases have become a mainstay of degron discovery (Table 1).

A good example for the evolution of degron research is the study of *Deg1* of matα2 in yeast. Structure prediction algorithms, combined with structure-function analyses of *deg1*, predicted the existence of an internal amphipathic helix within the degron that functions as a substrate recognition determinant by the E3 Ub ligase Doa10 [56]. The same motif is also present in other Doa10 substrates, including the non-coding *CL1* degron [31,35], the *DegAB* degron of the C-terminus of the yeast kinetochore protein Ndc10 [8] and the cadmium and copper-sensitive degron of the cadmium exporter, Pca1 [57]. An ultimate Confirmation of an amphipathic helix as a Doa10 recognition determinant was provided by Geffen and co-workers [39], who identified several Doa10 degrons, including a conserved element from the yeast glycolytic enzyme Enolase. Crystal structure of yeast enolase Eno1 [58] (PDB: 1EBG) indicated that the putative Eno degron is comprised of an amphipathic helix. Hydrophobic-to-polar mutation within this secondary structure inhibited Doa10-mediated degradation and confirmed that this secondary structure is a *bona fide* PQC degron [39].

In addition to an amphipathic helix (termed *DegA*), the Ndc10-derived *DegAB* degron also consists of a short C-terminal sequence (termed *DegB*), where a six-mer hydrophobic sequence is essential for proteasomal degradation, albeit redundant for Doa10-mediated ubiquitylation [59]. This finding demonstrates that E3-recognition may not be enough to induce proteasomal degradation and that degrons are structurally and functionally diverse.

Closer characterization of the *DegA* degron reveals that the hydrophobic surface of the helix is flanked by positively charged amino acids [8] a feature that also comprises a binding motif to Hsp70, implying chaperone requirement [60]. Indeed, Hsp70s were indispensable for the degradation of Deg*AB* as well as for every degron discovered by the Michaelis screen whether Doa10-dependent or not [37,61].

A major advantage of HTP screening over traditional degron search is that it results in a larger dataset that can be used for sorting explicit degron features. Indeed, this was key to discovering C-terminal degrons within the human peptidome [41,48] and consequently, to identifying cullin2 RING ligases (CRLs), specifically BC box adaptors, as their cognate E3 Ub ligases [41,48]. Interestingly, glycine residues are depleted from the carboxyl termini of eukaryotic proteins, suggesting a minor role in proteome regulation under standard growth conditions [41]. However, errors in protein translation that lead to premature termination, can destabilize the resulting aberrant proteins by positioning at the C-terminus an otherwise internal glycine [48]. Consequently, glycine-end degrons may function as fail-safe mechanism of PQC.

A crystal structure of the CRL2 substrate receptor, Kelch-Domain C2 (KLHDC2), in complex with C-end diglycine peptides revealed the mechanism of degron recognition by its adaptor. Apparently, a deep binding pocket at the surface of KLHDC2, accommodates glycine residues exclusively by excluding amino acid side chains [62]. The initial diglycine binding promotes additional interaction with C-terminal backbone carbonyl groups resulting in an extremely high affinity interaction (within a single digit nanomolar concentrations) [62].

The α amino acid at the N-terminus of proteins is recognized by the N-degron pathway [25]. Originally, E3α (Ubr1 in yeast) was identified as a cognate E3 ligase that recognizes specific N-terminal residues [23]. Two main branches of the N-degron pathway target proteins for proteasomal degradation have been discerned. One recognizing proteins with Nα acetylated proteins [63] and the other, an unmodified Nα, having basic or a bulky hydrophobic side chain [25,64]. Kats and co-workers, who investigated sequences appended at the N-terminus of a tested reporter, discovered that hydrophobicity was a major degron determinant recognized mostly by Doa10 [46]. The hydrophobic nature and the identity of Doa10 as their cognate E3 Ub ligase suggest that N-terminal degrons play an important role in PQC-mediated degradation, possibly when protein misfolding exposes otherwise buried N-end tails.

## 4. Additional Insights Obtained from Degron Research

High throughput degron screens, facilitated by NGS and coupled to data analysis by sophisticated bioinformatic algorithms, have become the pillars of contemporary degron discovery. Comprehensive degron screens thus not only have discovered numerous degradation determinants but have also provided novel insights into the complexity of degron mode of action and composition.

We now realize that the position of a degron within proteins determines its activity—there are N-terminal and C-terminal degrons with very little positional overlap [6]. We also became aware of spatial regulation—some degrons are exclusively active in the nucleus, others in the cytoplasm but most in both compartments [39]. Since proteasomes are present both in the nucleus and the cytoplasm [65,66], the spatial regulation of degron activity is obviously explained by the cellular distribution of their cognate E3 ligases. For example, many of the dual compartment-active degrons are substrates of Doa10 that resides in the yeast endoplasmic reticulum (ER)/nuclear membrane and degrades protein both in the nucleoplasm and the cytoplasm [67]. The San1 degrons are active only in the nucleus where the E3 resides [68], while Hrd1 degrons are recognized exclusively in the ER lumen [69]. Both the Koren and Lin screens defined cytosolic CRL-type E3s as the C-terminal degron recognition site of the Ub ligation system [41,48]. From these and other studies, we learned that the discovery potential of a single screen is limited by where in the cell degrons are expressed. Consequently, future degron screens must include expression of a single library in distinct cellular compartments. To this end, ER localization can be driven by addition of a signal peptide while nuclear localization or exclusion can be easily manipulated by adding or omitting nuclear localization signals to appended reporters as currently performed in our lab [39].

Cellular protein half-lives are extremely diverse, from several minutes to days. Obviously, degrons with varying potencies and surface exposure are involved. Identifying the full range of degron activity is therefore a prime objective. Achieving this goal has been substantially enhanced by the development of FACS-based methods and in particular, the GPS that rely on relative reporter fluorescence. Thus, these methods can detect a wide range of degron activities in a single experiment simply by analyzing the distribution of dsRed/GFP cell populations [41,48]

Yet, despite the abundance of data produced by state-of-the-art degron screens, it did not reveal, in most cases, comprehensive structure function correlations. Except for several incidents (amphipathic helix and C-terminal diglycine), most screens discovered degron sequences that apparently cannot be merely classified according to an obvious sequence or structural property. The use of complex synthetic libraries that facilitated discovery and definition of C- and N-terminal degrons [41,46], suggest that testing highly complex DNA libraries, possibly in a compartment-specific manner, will likely reveal these elusive relationships.

Beyond the scope of this review is a large group of Ub-independent degrons [70] that trigger degradation by either the 26S or 20S proteasome. The most familiar of these is ornithine decarboxylase that is targeted to the 26S proteasome via the auxiliary protein Antizyme [71]. But generally, the degrons and the mode of substrate recognition of Ub-independent proteasome-dependent proteolysis is obscure. Thus far, only a handful of Ub-independent degrons have been identified, nevertheless, HTP screening has not been applied to enhance further discovery. To discover Ub-independent degrons, HTP methods that eliminate Ub-dependent degrons must be designed. This can be accomplished, for example, by conducting screens in Ub-activating enzyme (E1)-inhibited cells.

## 5. Future Perspectives

Degron discovery projects, most of which have been conducted in yeast, provided important insights into principles by which intracellular protein degradation networks operate. However, the ultimate task is to understand how the human degronome is regulated in health and what goes awry in disease. The immensity of this challenge is acknowledged when one considers the much larger number of E3 ligases encoded in the human genome compared with that of yeast [72,73], indicating that the human degronome is exceedingly more complex than its yeast counterpart.

Albert Einstein once said, “The more I learn the more I realize how much I don’t know”. This sounds like a fair assessment of where we currently stand in terms of understanding how the yeast, let alone the human proteome, is regulated. Nevertheless, the realization of the unknown and the development of potent HTP cellular methods combined with powerful computational capacity, now makes the mission possible.

## Figures and Tables

**Table 1:** Summary of high throughput degron investigations.

Study	Key Findings	Reference
N-terminal degrons from short artificial PCR-based sequences. Method used: β-Gal-dependent blue/white assay on plates.	Three classes of degrons with considerably long half-lives: Ubr1-dependent N-degrons, Doa10 substrates and Ubc4/5-dependent short tracks of hydrophobic residues	[30]
C-terminal degrons from a truncated yeast genomic library. Method used: β-Gal-dependent blue/white assay on plates.	A Doa10 substrate. Does not code for an actual protein.	[31]
C-terminal degrons from a truncated yeast genomic library. Method used: Ura3-dependent growth on plates.	Nine Doa10 substrates, none code for an actual protein. Most are largely hydrophobic. Some contain an amphipathic helix.	[31]
C-terminal degrons from a truncated yeast genomic library. Method used: Ura3-dependent growth on plates.	Seventy-seven unique sequences, mostly Doa10, but also Ltn1 and few Ubr1/ San1 substrates. A principal role for Ydj1 and Ssa1/Ssa2 chaperones in substrate degradation.	[37]
C-terminal degrons from a truncated cDNA library. Method used: Ura3 competition assay in liquid media	Thousands of cytosolic and nuclear degrons, hundreds of them code for peptides within actual proteins. Multiple targets of Doa10. Nuclear localization changes degron propensity.	[39]
N-terminal synthetic degrons, present in the yeast proteome. Method used: tFT-Employing FACS to Separate degrons using mCherry and sfGFP as biological timers.	~26% of nascent protein N termini encode cryptic degrons. Most of them are Doa10 substrates. Hydrophobicity is the key feature for degradation. N-terminal acetylation and the N-degron pathway rarely function as a degrons.	[46]
C-terminal synthetic degrons, present in the human proteome. Method used: GPS- Employing FACS to Separate degrons based on the ratio RFP/GFP.	Distinct classes of C-degrons, mainly those enriched in Gly and Arg residues, are degraded by cullin-RING Ub E3 ligases. Mutant proteins with pre-mature stop codon are the principal substrates.	[41,48]
